# Optimal Environmental Policy for Heterogeneous Governments in China

**DOI:** 10.3390/ijerph20043087

**Published:** 2023-02-10

**Authors:** Ren Wang, Yuxiang Bian, Han Gao, Jie Hou

**Affiliations:** 1 Business School, East China University of Political Science and Law, Shanghai 201620, China; 2 International School of Economics and Management, Capital University of Economics and Business, Beijing 100070, China

**Keywords:** government type, pollution emission, optimal environmental policy

## Abstract

The purpose of the article is to study how the shift in the developing philosophy of China’s central leadership has impacted the management style of China’s local governments and, in turn, the country’s economic and environmental equilibrium. We use a real business cycle model with environmental variables and divide governments into those with/without environmental concerns and into those with long- and short-term policy horizons. We find that forcing local governments to plan in the long run is effective only when those governments are simultaneously mandated to consider the environment to be as important as the economy. Theoretical results show that both output and pollution levels are highest under governments without environmental obligations, intermediate under long-term governments with environmental obligations, and lowest under short-term governments with such obligations.

## 1. Introduction

The last decade has witnessed a major shift in China’s development philosophy. Since China ascended to the rank of the second largest economy in 2010, the country’s unified leadership has placed environmental protection foremost in its strategic thinking, on par with GDP growth. The administration has taken tangible and forceful measures to ensure the effective and nationwide implementation of this transition in the development mode. China’s president Xi has reiterated on multiple occasions, for example, at the 2018 National Conference on Ecological Environmental Protection, that party and government officials who jeopardize the environment will be held “permanently accountable”, enforcing lifetime responsibility tracking and indicating a willingness to take on local governing officials of all ranks for their actions during their term affecting environments.

The strong determination to implement this growth mode transition and its forceful execution from the top down have significant impacts on local leaders’ behaviors. The shift in the development philosophy of China’s central leadership has impacted the goals and management styles of China’s local governments and, in turn, the country’s economic and environmental equilibrium. According to the Environmental Protection Law of the People’s Republic of China, revised in 2014, the competent central environmental protection department implements unified supervision and management of national environmental protection work, while local governments should be responsible for the environmental quality of their administrative regions and implement unified supervision and management of environmental protection work. The specific responsibilities of local governments include increasing financial investment in protecting and improving the environment, preventing and controlling pollution and other public hazards, and strengthening publicity on environmental protection and popularizing the concept. This assignment of responsibilities has given rise to two interesting questions: How do local leaders with different objectives and timeframes choose policies to meet their targets? What is the ultimate effect of those policies on the environment and economy? The answers to these questions will have significant theoretical and pragmatic consequences both for the understanding of the relationship between governance behavior and economic and environmental well-being, and for the determination of the optimal strategy of China’s central and local governments to maintain a balance between economic development and environmental preservation. We propose the hypothesis that elevating environmental protection to the same level of priority as economic development and introducing lifetime tracking of local leading officials’ service records may significantly alter the equilibrium output and pollution emission levels under their authority. This paper aims to provide some insights into these issues and test this hypothesis.

We constructed a real business cycle (RBC) model to study the optimal environmental policies and the resulting economic output and pollution emissions under different types of government. We categorized governments on the basis of two sets of indicators: short-term and long-term governments and governments with and without environmental concerns. We hence assumed a total of four types of governments. For each type of government, we explored the optimal environmental taxes and subsidies, as these are the two most commonly adopted policy tools at the disposal of China’s local governments, which provide monetary rewards (subsidies) or penalties (taxes) to enterprises for the environmental impact of their operational activities and thereby induce firms to adopt production decisions that are friendlier to the environment. According to the Environmental Protection Tax Law of the People’s Republic of China, a local government may propose the determination of and adjustment to the applicable tax amounts of taxable pollutants within a range of taxable amounts by giving full consideration to local environmental capacity and the requirements of economic development objectives. In addition to a pollution tax, local governments subsidize polluters to reduce emissions. Environmental subsidies are forms of financial government support for activities believed to be environmentally friendly. Local governments award various subsidies to help firms improve their environmental protection equipment and environmental protection processes. We compare the efficiency of environmental policies and their effects on production and pollution output under long- and short-term local governments and the behavior of local governments under assessment standards that prioritize economic performance and standards that balance economic and environmental performance.

We found the following results: without the decree by the central leadership that local administrations will be judged by their performance in supporting environmental preservation in addition to economic growth, forcing local cadres to plan with a long horizon does not have an effect on the final equilibrium of the output and pollution levels. Only for a government with environmental obligations to fulfill is the policy choice (and thus the resulting equilibrium) impacted by whether it espouses a long- or short-term policy horizon; long-term governments show higher levels of both output and pollution emission. Therefore, when an administration has environmental objectives to achieve, governments with different planning horizons will indeed espouse distinct policy sets that lead to different equilibrium levels of output and pollution emissions. On the other hand, the policy choice of a government focusing only on economic achievement does not depend on whether the policy horizon is long- or short-term. In this regard, ignoring the type of government under study may produce biased estimates of its policy impacts on the economy.

To the best of our knowledge, this paper represents the first attempt to pursue this avenue of inquiry and offers the first findings relevant to practice. There are empirical studies [1,2,3,4,5] documenting that the environmental deterioration in China in the late 20th century and early 21st century is due to economic growth being the top priority recognized by local authorities, which causes many cadres to focus on economic development at the expense of the environment. As the central government gradually increases the assessment of environmental performance, a dual-track system of economic development and environmental governance is gradually replacing the previous system. Local leaders must in turn increase the weight of environmental quality relative to that placed on economic development [6]. The conclusions of our model provide a theoretical explanation for the relationship between the transformation of the local government’s objective function and the dynamic changes in local economic growth and environmental quality found in related empirical studies. The optimal behavior of local administration depends on the government’s type (objective function). Our findings are thus consistent with those of the aforementioned empirical studies.

The rest of the paper is organized as follows. Section 2 reviews the related literature. Section 3 introduces the model settings. Section 4 presents the different types of governments and their optimal environmental policies. Section 5 reports on the numerical analysis. Concluding remarks are provided in Section 6.

## 2. Literature Review

Previous studies have shown that local governments in China face a tradeoff between economic growth and environmental protection [7,8,9]. Under the old system that assessed local ruling officials’ performance primarily on the basis of their economic achievements, officials focused on projects that best advanced the local economy under their remit within their expected tenure to maximize their chances of promotion upon completing their term [10,11,12,13]. Strict environmental policies from the top down could often receive selective implementation or even outright opposition from local leadership [14], which is responsible for the ultimate execution of such policies [15,16,17]. In addition, it is argued that fiscal decentralization gives local governments an incentive to pay exclusive attention to the economy to increase their tax revenue [18,19,20,21].

In recent years, the appraisal and evaluation standards for officials’ performance and promotion have been fundamentally updated to reflect the central authority’s shift in development focus [22,23,24]. Ranking officials are tracked and held responsible for life for the consequences of their governing actions on the environment. Starting in 2014, the Ministry of Ecology and Environment of China began to conduct warning talks with local government officials who failed to fulfill their environmental protection duties [25,26,27]. Such measures have influenced local governments to adopt policies that stress environmental improvement along with economic prosperity, with their goals not confined to their term in power but spanning a much longer horizon [23,28,29]. Several studies [30,31] have documented the importance of governance in reducing pollution emissions. They find that effective governance systems can help significantly develop the clean energy industry and reduce carbon dioxide emissions. However, a political climate emphasizing environmental regulation may hinder economic development [32,33].

Regarding the dynamic stochastic general equilibrium (DSGE) approach used in this paper, there is a growing body of literature that examines the effect of environmental policy on the economy using environmental DSGE (EDSGE) (a DSGE model with environmental variables) and, particularly, RBC models [34,35,36,37,38,39,40]. Nevertheless, most existing work focuses on analyzing separate environmental policies and their implications while paying less attention to the authority issuing the policies and the combined effect of such policies on both the economy and the environment. The general EDSGE framework that we adopted in this research is distinguished from previous models by its inclusion of different types of governments. We then theoretically examined the impact of government type on output and pollution emissions.

## 3. The Model

The model was built on that of [35]. We incorporated different government types and analyzed the optimal environmental policy and the equilibrium output and pollution emission levels under different policy objective functions.

### 3.1. Households

The representative household derives utility from consumption $C_{1,t}$, and disutility from labor supply $N_{t}$, and attempts to maximize its expected long-term utility:

(1) $$E_{t}\sum_{t=0}^{\infty }\beta^{t}\left(\log C_{t}-\chi N_{t}\right)$$

where β is the household discount factor and χ is the labor preference parameter.

In period *t*, the household receives gross interest income on deposits $D_{t-1}R_{t-1}$ and wage income $W_{t}N_{t}$. The household also makes new deposits $D_{t}$ and purchases goods $C_{t}$ with the price normalized to 1. The budget constraint for the household is given by the following:

(2) $$\begin{array}{c} D_{t}+C_{t}=D_{t-1}R_{t-1}+W_{t}N_{t} \end{array}$$

The first-order conditions with respect to $C_{t}$, $N_{t}$, and $D_{t}$ are as follows:

(3) $$\lambda_{t}=C_{t}^{-1}$$

(4) $$W_{t}\lambda_{t}=\chi$$

(5) $$1=\beta E_{t}\frac{\lambda_{t+1}}{\lambda_{t}}R_{t}$$

Here, $\lambda_{t}$ is the Lagrange multiplier for the budget constraint. Equations (3)–(5) provide the households’ optimal behavior of consumption and labor supply.

### 3.2. The Goods Producer

In a perfectly competitive market, the producer of goods hires $N_{t}$ to produce a consumption good $Y_{t}$ according to the following production function:

(6) $$Y_{t}=\left[1-d\left(x_{t}\right)\right]A_{t}N_{t}$$

where *d* is an increasing function that takes values between 0 and 1 and represents the loss of potential output from the pollution stock $x_{t}$, following the dynamic integrated model of climate and the economy (DICE) setting in [35,41].

(7) $$d\left(x_{t}\right)=d_{2}x_{t}^{2}-d_{1}x_{t}+d_{0}$$

The pollution stock decays at a linear rate equal to η.

(8) $$x_{t}=\eta x_{t-1}+EM_{t}+EM_{t}^{w}$$

Here, $EM_{t}^{w}$ is the current-period pollution emissions from the rest of the world, and domestic pollution emissions $EM_{t}$ are assumed to be proportional to output, as in [35,36,37] (there are also studies [42,43,44,45,46,47,48,49] in which higher levels of energy consumption lead to greater economic activity and stimulate pollution emissions):

(9) $$EM_{t}=\left(1-ER_{t}\right)\psi Y_{t}$$

where ψ is the pollution emissions coefficient and $ER_{t}$ is the proportion of pollution emissions reduction, $ER_{t}\in \left(0,1\right)$. The cost of emission abatement is

(10) $$CO_{t}=vER_{t}^{u}Y_{t}$$

where v>0 and u>1 are technological parameters of the abatement cost. The goods producer needs to pay the government a tax τ for each unit of pollution emissions.

The goods producer maximizes its profit:

(11) $$\Pi_{t}=Y_{t}-W_{t}N_{t}-\tau EM_{t}-\left(1-z\right)CO_{t}$$

where 0<z<1 is the environmental subsidy. Both the environmental tax $\tau_{t}$ and subsidy $z_{t}$ are key policy tools for the local government to maximize its objective function. The optimality of $N_{t}$ and $ER_{t}$ requires:

(12) $$W_{t}+\tau \frac{EM_{t}}{N_{t}}+\left(1-z\right)\frac{CO_{t}}{N_{t}}=\frac{Y_{t}}{N_{t}}$$

(13) $$ER_{t}={\left(\frac{\tau \psi }{uv(1-z)}\right)}^{\frac{1}{u-1}}$$

 **Proposition 1.** *A higher environmental tax or subsidy rate leads to a larger proportion of pollution emission reduction. (Proof: $\frac{\partial ER_{t}}{\partial z}>0$, $\frac{\partial ER_{t}}{\partial \tau }>0$).*

### 3.3. Government

The flow budget constraint of the government is simply

(14) $$\tau EM_{t}=G_{t}+zCO_{t}$$

where $\tau EM_{t}$ is government tax revenue, $G_{t}$ is government expenditure, and $zCO_{t}$ represents government subsidies.

### 3.4. Equilibrium

Equilibrium in the goods market requires that

(15) $$Y_{t}=C_{t}+G_{t}+CO_{t}$$

Together with Equations (3), (4), (6), (12) and (14), output can be solved for as follows:

(16) $$Y_{t}=\frac{A_{t}}{\chi }\left[1-d\left(x_{t}\right)\right]$$

Therefore, labor supply can be derived as

(17) $$N_{t}=\frac{1}{\chi }\left[1-d\left(x_{t}\right)\right]$$

## 4. Type of Government

We considered four types of governments, namely, short-term and long-term governments with and without environmental concerns. We regard horizons of less than or equal to one term (usually 5 years) as short-term (one-term accountable) and those of more than 5 years as long-term (permanently accountable).

### 4.1. Government without Environmental Concerns

#### 4.1.1. Short-Term Government

A short-term (shortsighted) government without environmental concerns cares only about maximizing current output $Y_{t}$ within its tenure. This relates to the period when China was low in absolute and per capita GDP and undergoing rapid economic expansion, and when the central leadership evaluated officials’ performance only by their achievements in power.

By using Equations (7) and (16), we can solve for the optimal pollution emission stock as follows:

(18) $$x^{*}=\frac{d_{1}}{2d_{2}}$$

#### 4.1.2. Long-Term Government

For various reasons, officials may consider developments of the region under their authority beyond their appointed term. This type of government, a long-term (farsighted) government without environmental concerns, aims to maximize the sum of the present value of current and future output $E_{t}\sum_{n=0}^{\infty }\beta^{n}Y_{t+n}$.

With Equations (7) and (16), the optimal pollution emission stock is the same as that under the short-term government without environmental concerns:

(19) $$x^{*}=\frac{d_{1}}{2d_{2}}$$

 **Proposition 2.** For both short- and long-term governments without environmental concerns, the objective of environmental policies is to achieve the lowest loss of potential output from pollution reduction. $(Proof:\frac{\partial d(x_{t})}{\partial x_{t}}{|}_{x^{*}}=0$, $\frac{\partial^{2}d\left(x_{t}\right)}{\partial x_{t}^{2}}{|}_{x^{*}}>0).$

In the steady state, where $x_{t}=x_{t-1}$, for both types of government without environmental concerns, Equations (8), (9) and (16) derive the equation that the optimal environmental tax τ and subsidy *z* must satisfy:

(20) $$\begin{array}{c} \tau ={\left(1-\frac{\left(1-\eta \right)x^{*}-EM^{w}}{\psi \frac{A}{\chi }\left[1-d\left(x^{*}\right)\right]}\right)}^{u-1}\frac{uv(1-z)}{\psi } \end{array}$$

### 4.2. Government with Environmental Concerns

#### 4.2.1. Short-Term Government

A short-term government with environmental concerns maximizes a weighted summation of the output and pollution levels:

(21) $$Y_{t}-\phi EM_{t}$$

where ϕ is the weight assigned to pollution emissions. Together with Equations (7), (8) and (16), the maximization problem leads to the following:

(22) $$\begin{array}{c} \frac{\partial Y_{t}}{\partial EM_{t}}=\phi >0 \end{array}$$

 **Proposition 3.** *Output is an increasing function of pollution emissions, and the marginal output of pollution emissions is determined by the weight assigned to pollution emissions in the objective function of the short-term government with environmental concerns.*

In the steady state, where $x_{t}=x_{t-1}$, Equations (9), (16) and (22) yield the optimal environmental tax and subsidy:

(23) $$\begin{array}{c} \tau ={\left(1-\frac{\left(1-\eta \right)x^{**}-EM^{w}}{\psi \frac{A}{\chi }\left[1-d\left(x^{**}\right)\right]}\right)}^{u-1}\frac{uv(1-z)}{\psi } \end{array}$$

where $x^{**}=\frac{d_{1}}{2d_{2}}-\frac{\phi \chi }{2d_{2}A}$.

#### 4.2.2. Long-Term Government

The recently established lifetime environmental responsibility system prompts officials in charge to think and act on the long horizon during their term. A long-term government with environmental concerns strives to maximize the weighted discounted summation of the output and pollution levels.

(24) $$E_{t}\sum_{n=0}^{\infty }\beta^{n}\left(Y_{t+n}-\phi EM_{t+n}\right)$$

Similar to Equations (22) and (23), we obtain the following:

(25) $$\begin{array}{c} \frac{\partial Y_{t}}{\partial EM_{t}}=\frac{\phi }{1+\beta \eta }>0 \end{array}$$

In the steady state, where $x_{t}=x_{t-1}$, Equations (9), (16) and (22) also yield the optimal environmental tax and subsidy:

(26) $$\begin{array}{c} \tau ={\left(1-\frac{\left(1-\eta \right)x^{***}-EM^{w}}{\psi \frac{A}{\chi }\left[1-d\left(x^{***}\right)\right]}\right)}^{u-1}\frac{uv(1-z)}{\psi } \end{array}$$

where $x^{***}=\frac{d_{1}}{2d_{2}}-\frac{\phi \chi }{2d_{2}A\left(1+\beta \eta \right)}$.

 **Proposition 4.** When a government is obligated to achieve both environmental protection and economic growth, whether it assumes a short- or long-term policy horizon impacts the final equilibrium. Compared to a short-term government, a long-term government can achieve a larger pollution emission stock (proof:$\;x^{***}>x^{**})$, more pollution emissions in each period (proof:$\;\frac{\partial EM}{\partial x}>0)$, higher output (proof:$\;\frac{\partial Y}{\partial x}>0)$, and a lower marginal output of pollution emissions (proof:$\;\phi >\frac{\phi }{1+\beta \eta })$.

In the tradeoff between economic growth and pollution emission, a long-term government can take advantage of the decay of the pollution stock to optimally choose a higher level of pollution emission and economic growth path over a longer time span than the short-term government.

 **Proposition 5.** *We find that for both short- and long-term governments with or without environmental concerns, there is a negative linear relationship between the optimal environmental tax rate and the optimal environmental subsidy rate. Therefore, these variables are substitutes for each other (proof: Equations (20), (23) and (26)).*

This shows that when the government maximizes its objective function, it can not only select a single policy, such as an environmental tax or environmental subsidy, but also choose a linear combination of the two. Environmental taxes restrain enterprises’ pollution emissions by increasing their pollution costs, while environmental subsidies stimulate enterprises’ emission reduction efforts by compensating them for their emission reduction costs. The linear relationship between the two can greatly improve the flexibility of government policy choices.

## 5. Numerical Analysis

We calibrated the model parameters based on existing research (we also tested different parameter values within a reasonable range, finding that the results of the model are robust). For example, we set the household discount factor β to 0.98267, in line with [35]. All the parameter values and sources are listed in Table 1. According to the Wind database, China was responsible for approximately 30.9% of global anthropogenic carbon emissions in 2020, so $EM^{w}$ was set at 2.24 times the steady-state value of EM.

**Figure 1:**
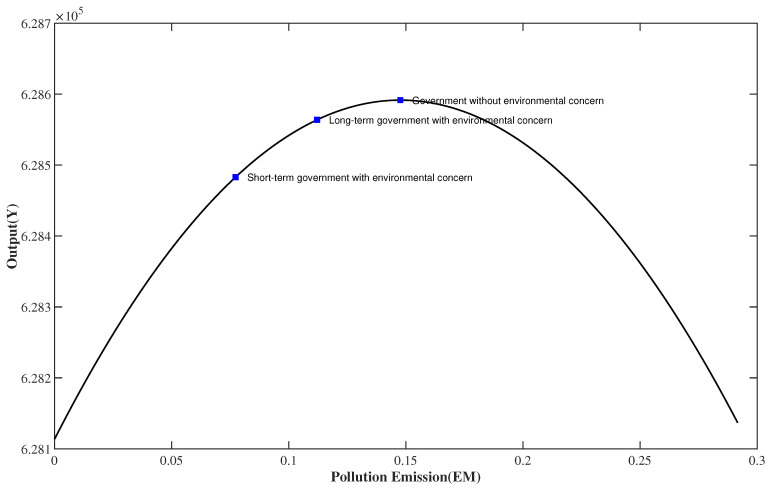
Optimal output and pollution emission combinations.

Figure 1 shows possible output and pollution emissions combinations for a local government. The government chooses the corresponding combination of environmental policies to maximize its objective function. It is worth noting that although the relationship between the optimal output and pollution levels shown in Figure 1 is an inverted U-shaped curve, a rational government stays on the left side of this curve, where the marginal output from pollution emissions is positive.

In this regard, our model provides a curve similar to the environmental Kuznets curve (EKC) but emphasizes the negative externalities of pollution emissions. The right side of the inverted U-shaped curve in Figure 1 shows that negative externalities caused by severe environmental pollution seriously damage production, resulting in a decline in output. The EKC hypothesis states that in the early stages of economic growth, pollution emissions increase and environmental quality declines but that beyond some level of per capita income, the trend reverses, so that at high income levels, economic growth leads to environmental improvement. This implies that with economic growth and the improvement in per capita income, economic society imposes higher requirements for environmental quality, thus reducing environmental pollution.

For a government focusing exclusively on economic growth, whether shortsighted or farsighted, environmental policy is only a tool to maximize output; that is, to reduce the negative impact of pollution emissions on output. Governments caring about green GDP face a tradeoff between economic growth and environmental protection. Environmental policies can be used to locate a balance between maximizing output and minimizing pollution emissions for such a government. Because the pollution stock decays over time, a long-term government concerned about green GDP optimally chooses higher pollution emissions and output in each period than one focusing on short-term green GDP. It does so by adopting a lower environmental tax rate and/or higher environmental subsidy rate in each period. The goods producer optimally adjusts its output and pollution emissions. The optimal choices of the different types of government are plotted in the graph.

Each point in Figure 1 can represent an optimal choice of the local government. The different means of environmental governance of the central government and the different environmental emphases of the localities make different types of governments choose different points. This highlights the importance of environmental governance to environmental pollution and economic growth.

## 6. Conclusions

Although it has long been known that China’s local governments face a tradeoff between environmental preservation and economic growth, the country’s recent switch in development principles from an exclusive focus on economic prosperity to a dual emphasis on economic and environmental performance has made the study of the relationship between government type and horizon and the equilibrium output and environmental status practical and urgent. This paper is among the first to theoretically explore this aspect with an RBC model. Four types of governments and their respective decisions and impacts on the equilibrium levels of output and pollution emissions are considered. We validate our hypothesis and answer the aforementioned research questions. The type of local government plays an important role in shaping environmental policies, and thus in determining the level of local pollution emissions. As expected, pollution emission capacity increases with an expansion of output. More interestingly, we record that both pollution emissions and output levels reach their highest levels under governments without environmental concerns and their lowest levels under a short-term government with environmental concerns, while the output and pollution emission levels under a long-term government with environmental concerns fall in the middle.

Our results explain the impact of two of the most pronounced decisions from China’s central leadership in the last decade, namely, elevating the priority of environmental protection to the same level as that of economic development and introducing lifetime tracking of local leading officials’ service records, on the behavior of its various local governments and ultimately on the general equilibrium output and pollution emission levels under their authority. Moreover, we show that lifetime service tracking and measures to force local leaders to think in the long run are impactful only with an emphasis on environmental protection. Output and pollution reach their highest levels when a local administration does not have environmental concerns, regardless of its planning horizon. At the same time, a long-term government with environmental concerns achieves intermediate levels on both outcomes, below those achieved by any government without environmental concerns and above those achieved by a short-term government with such concerns. This speaks to the importance of principles of good governance (such as accountability) for the environment. When environmental issues are taken as an important variable in the assessment of local leaders’ governance, local governments’ attention to environmental issues and enthusiasm for environmental protection increase significantly, helping reduce local pollution emissions and improve environmental quality.

These findings shed light on how to balance environmental preservation and economic development and have urgent practical implications. They call for the central government to pay attention to agency problems when developing a green economy. The central government must improve its environmental governance by aligning the interests of local leaders with environmental protection by adjusting the promotion mechanisms and tenure responsibilities of local leaders. Only in this way can the environment be effectively protected. For example, the ”river chief system”, which requires government officials at all levels to take full responsibility for the protection of water resources in the areas under their jurisdiction, greatly improves the ability to prevent and control water pollution.

Our model is a simple RBC model that does not take into account the stickiness of prices and wages or the effect of capital adjustment costs on output and pollution emissions. Therefore, some further research avenues remain to be explored; for example, by means of a new Keynesian model with heterogeneous governments or a game theory analysis of the interaction between the central and local governments, and the competition among local governments, in line with [50].

## Figures and Tables

**Table 1:** Parameter calibration.

Par.	Value	Source	Description
β	0.98267	[35]	Household discount factor
η	0.9979	[35]	Pollution decay
$d_{2}$	$1.4647\times 10^{-8}$	[35,41]	Parameter of the damage function
$d_{1}$	$6.6722\times 10^{-6}$	[35,41]	Parameter of the damage function
$d_{0}$	$1.3950\times 10^{-3}$	[35,41]	Parameter of the damage function
*A*	1.2480	[37]	Total factor productivity
*v*	0.185	[37]	Abatement cost function coefficient
*u*	2.8	[37]	Abatement cost function parameter
ψ	0.45	[37]	Emissions per unit of output
χ	19.8413	[37]	Disutility of labor

## Data Availability

Not applicable.

## Abbreviations

The following abbreviations are used in this manuscript:

RBC	real business cycle model
DSGE	dynamic stochastic general equilibrium
EDSGE	environmental dynamic stochastic general equilibrium
DICE	dynamic integrated model of climate and the economy

## Supplement: RBC Model

The best-known paper in the real business cycle (RBC) literature is by [51]. They introduce both a specific theory of business cycles and a methodology for testing competing theories of business cycles.

The RBC model has several characteristics. For example, the economy should always be modeled using dynamic general equilibrium models, money is of little importance in business cycles, business cycles are created by agents with rational expectations responding optimally to real shocks, and a model’s suitability for describing reality should be evaluated using a quantitative technique known as “calibration”.

A more detailed introduction can be found in [52].
